# Tamoxifen differentially modulates endometrial hyperplasia via wild-type and mutant p53 regulation of the ALKBH5-REG1A axis

**DOI:** 10.3389/fonc.2026.1784356

**Published:** 2026-03-19

**Authors:** Rencheng Wang, Jianhua Ji, Lei Liu

**Affiliations:** 1 Department of Obstetrics and Gynecology, Renhe Hospital, Shanghai, China; 2 Department of Obstetrics and Gynecology, Obstetrics & Gynecology Hospital of Fudan University, Shanghai Key Lab of Reproduction and Development, Shanghai Key Lab of Female Reproductive Endocrine Related Diseases, Shanghai, China

**Keywords:** ALKBH5, endometrial hyperplasia, p53, REG1A, tamoxifen

## Abstract

**Introduction:**

Tamoxifen is a cornerstone of endocrine therapy for estrogen receptor–positive breast cancer; however, its partial estrogen agonist activity in the endometrium predisposes patients to hyperplasia and, in some cases, malignant transformation. The molecular mechanisms underlying this tissue-specific adverse effect remain incompletely understood.

**Methods:**

We employed immortalized human endometrial epithelial cells to investigate the role of p53 in tamoxifen-induced proliferation. Cells were genetically manipulated to express wild-type (WT) or mutant p53 (R248Q), and ALKBH5 or REG1A was silenced or overexpressed using lentiviral constructs. A comprehensive set of molecular techniques-including quantitative reverse transcription PCR (qRT-PCR), Western blotting, chromatin immunoprecipitation (ChIP), luciferase reporter assays, methylated RNA immunoprecipitation (MeRIP), RNA immunoprecipitation (RIP), and functional proliferation assays (CCK-8 and colony formation)-was applied to dissect transcriptional and post-transcriptional regulatory mechanisms.

**Results:**

Tamoxifen promoted the recruitment of WT p53 to the ALKBH5 promoter, transcriptionally activating this m^6^A RNA demethylase. ALKBH5 subsequently erased m^6^A modifications from REG1A mRNA, preventing YTHDF2-mediated decay and thereby stabilizing REG1A expression. Elevated REG1A protein functioned as a negative feedback regulator, attenuating tamoxifen-induced proliferation. In stark contrast, the p53 R248Q mutant, despite retaining promoter-binding capacity, suppressed ALKBH5 transcription-potentially through altered cofactor recruitment-leading to increased m^6^A methylation of REG1A transcripts, enhanced YTHDF2-dependent degradation, and consequently, exaggerated cellular proliferation. Loss-of-function and genetic rescue experiments established that ALKBH5 is both necessary and sufficient to regulate REG1A mRNA stability, and that REG1A serves as the critical downstream effector mediating proliferative restraint under tamoxifen treatment.

**Conclusions:**

Tamoxifen’s anti-proliferative effects in endometrial epithelial cells are critically dependent on WT p53, which coordinates a protective epitranscriptomic regulatory axis. In contrast, mutant p53 disrupts this checkpoint and redirects tamoxifen signaling toward hyperproliferation. These findings establish a mechanistic link between hormonal signaling, p53 allelic status, and m^6^A-dependent post-transcriptional regulation. Although further *in vivo* validation is required, disruption of the ALKBH5–REG1A axis may contribute to heterogeneous endometrial responses to tamoxifen, thereby providing a conceptual framework for biomarker-oriented investigation.

## 1 Introduction

Tamoxifen (TAM) was the first selective estrogen receptor modulator (SERM) approved by the United States Food and Drug Administration for adjuvant endocrine therapy in breast cancer. Globally, breast cancer remains the most common malignancy among women (1, 2). According to the National Comprehensive Cancer Network (NCCN) guidelines, tamoxifen is recommended for 5 years as adjuvant treatment in premenopausal patients with estrogen receptor (ER)-positive invasive breast cancer or ductal carcinoma *in situ* (3). Women who remain premenopausal after the initial treatment may continue tamoxifen for an additional 5 years, whereas postmenopausal patients may either extend tamoxifen for 5 years or switch to an aromatase inhibitor (3). While tamoxifen antagonizes estrogen signaling in breast tissue, it exerts estrogen-like effects in the endometrium, raising concerns about its tumor-promoting potential and associated adverse outcomes. Clinical studies have consistently reported an increased risk of uterine disorders-including endometrial polyps, hyperplasia, carcinoma, and other uterine malignancies-particularly among postmenopausal women receiving tamoxifen (4). Furthermore, a meta-analysis of 55 randomized clinical trials confirmed not only a higher incidence of endometrial cancer but also increased endometrial cancer–related mortality in postmenopausal patients treated with tamoxifen as adjuvant therapy for early breast cancer (5). Despite these observations, the molecular mechanisms underlying tamoxifen-induced endometrial hyperplasia remain largely undefined, and effective preventive or therapeutic strategies are still lacking.

One key candidate regulator is the tumor suppressor p53, which plays a central role in maintaining genomic stability and restraining aberrant proliferation. Wild-type p53 responds to genotoxic and oncogenic stress by inducing cell cycle arrest, apoptosis, or senescence, thereby preventing malignant transformation (6). In contrast, mutations in p53 not only abolish its protective functions but can also confer gain-of-function (GOF) properties that promote cell survival, metabolic reprogramming, and therapy resistance (7, 8). In the endometrium, immunohistochemical studies have shown that wild-type p53 is rarely altered in simple hyperplasia, whereas abnormal p53 expression or mutations are increasingly detected in atypical hyperplasia and endometrial carcinoma, linking p53 dysfunction to disease progression (9, 10). Importantly, the functional divergence between wild-type and mutant p53 also affects responses to endocrine therapy. Fernandez-Cuesta et al. demonstrated that breast cancer cell lines harboring mutant p53 were resistant to the cytotoxic effects of 4-hydroxytamoxifen, whereas wild-type p53 lines exhibited reduced proliferation under similar treatment (11). Moreover, molecular analyses suggest that mutant p53 can reprogram transcriptional networks in ways that may override tamoxifen’s antiproliferative effects (12). Collectively, these findings raise the possibility that p53 status critically shapes tamoxifen’s actions in the endometrium, with wild-type p53 potentially exerting protective effects while mutant p53 may facilitate endometrial hyperplasia and progression. This dichotomy highlights a major gap in understanding how tamoxifen interacts with p53-dependent pathways in the uterine epithelium-a gap that the present study seeks to address.

Recent studies have increasingly implicated N6-methyladenosine (m^6^A) messenger RNA methylation and its modulators in the pathophysiology of endometrial diseases (13, 14). Alpha-ketoglutarate-dependent dioxygenase alkB homolog 5 (ALKBH5), one of the major m^6^A demethylases (“erasers”), has been shown to take part in the modulation of m^6^A modification and controls various cell processes. ALKBH5-mediated m^6^A demethylation regulates gene expression by affecting multiple events in RNA metabolism, e.g., pre-mRNA processing, mRNA decay and translation (15). Mounting evidence shows that ALKBH5 plays critical roles in a variety of human malignancies, mostly via post-transcriptional regulation of oncogenes or tumor suppressors in an m^6^A-dependent manner (15–17). In particular, Pu et al. demonstrated that ALKBH5 demethylates m^6^A modifications on IGF1R mRNA, thereby increasing its stability and promoting downstream signaling, ultimately enhancing endometrial cancer cell proliferation and invasion (18). Complementing this evidence, other studies have shown that ALKBH5 promotes disease progression by enhancing lncRNA UBOX5-AS1 expression through m^6^A demethylation, thereby facilitating autophagy, cell proliferation, migration, and invasion in ovarian endometriosis (19). Moreover, ALKBH5 is downregulated in intrauterine adhesion and plays a critical role in regulating endometrial fibrosis through FABP4 mRNA m^6^A methylation and lipid metabolism (20). Collectively, these findings highlight ALKBH5 as a key regulator of endometrial pathophysiology, underscoring its involvement in the progression of endometrium-related diseases.

REG1A (Regenerating Islet-Derived 1 Alpha), also known as pancreatic stone protein (PSP), is a member of the Reg gene family, which is involved in tissue regeneration and inflammation (21). In various malignancies, particularly colorectal cancer, REG1A expression is significantly elevated and correlates with advanced disease stage, lymph node metastasis, and peritoneal carcinomatosis, suggesting its role in tumor progression (22, 23). Functionally, REG1A promotes cell proliferation, migration, and invasion by modulating metabolic and signaling pathways, including the β-catenin/MYC/LDHA axis, which contributes to glycolytic reprogramming in CRC cells (24). Beyond colorectal cancer, REG1A has also been implicated in gastrointestinal tumorigenesis and has been shown to accelerate pancreatic cancer progression, particularly in patients with diabetes (25). In gastric cancer, IL-6/STAT3 signaling induces REG1A expression, conferring anti-apoptotic properties that support malignant transformation and tumor growth (26). Collectively, these findings highlight REG1A as a potent oncogenic effector that integrates cell cycle regulation with metabolic reprogramming to promote cancer progression across multiple organ systems.

Despite these advances, the mechanistic interplay between tamoxifen signaling, p53-dependent transcriptional regulation, and epitranscriptomic modulation by ALKBH5, together with the oncogenic activity of REG1A in the uterine epithelium, remains poorly understood. Current evidence suggests that these pathways may converge to influence the proliferative and survival capacity of endometrial cells, particularly under conditions of hormonal perturbation. However, whether tamoxifen-induced alterations in p53 activity intersect with ALKBH5-mediated m^6^A dynamics and REG1A-driven proliferative signaling has not been systematically explored. To address this knowledge gap, the present study aims to delineate the functional crosstalk among tamoxifen, p53, ALKBH5, and REG1A, thereby uncovering novel molecular mechanisms underlying endometrial pathology and identifying potential therapeutic targets.

## 2 Materials and methods

### 2.1 Cell culture

Immortalized human endometrial epithelial cell (hEEC) lines, including EM-E6/E7/TERT, EM-PR, EM-E6/E7/TERT/PRA, and EM-E6/E7/TERT/PRA/PRB^+^, were used in this study. The parental EM-E6/E7/TERT line was originally generated by immortalizing primary hEECs using HPV-E6/E7 and hTERT, as previously described (27, 28). Cells were maintained in DMEM/F12 (1:1) medium (Gibco), supplemented with 10% fetal bovine serum (Gibco) and 1% penicillin-streptomycin, at 37 °C in a humidified incubator with 5% CO_2_. Cells were subcultured at approximately 80% confluence using 0.25% trypsin-EDTA.

### 2.2 Lentiviral constructs, viral packaging, and establishment of stable cell lines

Full-length cDNAs encoding human wild-type p53 (TP53-WT), mutant p53 (TP53-R248Q), and ALKBH5 were subcloned into the pLV-EF1α-puro lentiviral overexpression vector. For loss-of-function studies, short hairpin RNA (shRNA) oligonucleotides targeting ALKBH5 or REG1A were designed and synthesized by Shanghai GenePharma Co., Ltd. and inserted into the pLKO.1-puro backbone to generate lentiviral knockdown constructs. Lentivirus production was performed in HEK293T cells by co-transfecting the transfer vector with psPAX2 packaging plasmid and pMD2.G envelope plasmid (Addgene) using Lipofectamine™ 3000 (Thermo Fisher Scientific), following the manufacturer’s protocol. Viral supernatants were collected at 48 and 72 hours post-transfection, cleared by centrifugation, filtered through 0.45-μm filters, and immediately applied to human endometrial epithelial cells in the presence of 8 μg/mL polybrene to enhance infection efficiency. Infected cells were selected with puromycin (2 μg/mL) for 5–7 days until all uninfected cells were eliminated. The efficiency of ALKBH5 or REG1A knockdown and p53 or ALKBH5 overexpression was confirmed by quantitative RT-PCR and Western blot prior to downstream functional assays.

### 2.3 Tamoxifen treatment

To model tamoxifen stimulation, cells were treated with 4-hydroxytamoxifen (4-OHT; Sigma-Aldrich), which was dissolved in ethanol to prepare a 10 mM stock solution and stored at −20 °C protected from light. For all experiments, 4-OHT was added to the culture medium at a final concentration of 1 μM and incubated for 48 hours. Vehicle controls received an equivalent volume of ethanol (final concentration <0.1%). For proliferation-related assays, 4-OHT treatment was initiated after lentiviral infection and subsequent puromycin selection, ensuring that p53, ALKBH5, and REG1A manipulations were stably established prior to drug exposure.

### 2.4 Quantitative real-time PCR

Total RNA was extracted from cultured cells using TRIzol reagent (Invitrogen) following the manufacturer’s protocol. RNA concentration and purity were determined using a NanoDrop 2000 spectrophotometer (Thermo Fisher Scientific), and samples with an A260/A280 ratio between 1.8 and 2.0 were used for subsequent analyses. One microgram of total RNA was reverse-transcribed into cDNA using the PrimeScript™ RT Master Mix (Takara) according to the manufacturer’s instructions. Quantitative real-time PCR was performed using TB Green Premix Ex Taq™ (Takara) on a QuantStudio 6 Flex Real-Time PCR System (Applied Biosystems). The reaction conditions were as follows: initial denaturation at 95 °C for 30 s, followed by 40 cycles of denaturation at 95 °C for 5 s and annealing/extension at 60 °C for 30 s. Gene expression levels were calculated using the 2^−^ΔΔCt method, with GAPDH serving as the endogenous control for normalization. Each experiment was performed using three independent biological replicates, and each sample was analyzed in technical duplicate to ensure data reliability.

### 2.5 Western blot analysis

Cells were lysed in RIPA buffer supplemented with protease and phosphatase inhibitors, incubated on ice for 30 minutes, and cleared by centrifugation at 12,000 × g for 15 minutes. Protein concentrations were quantified using a BCA Protein Assay kit (Thermo Fisher). Equal amounts of protein (20-30 μg) were separated via SDS-PAGE and transferred to PVDF membranes using a semi-dry transfer system. Membranes were blocked with 5% BSA in TBST for 1 h at room temperature and incubated overnight at 4 °C with primary antibodies against ALKBH5 (1:1000; Cat. No. ab195377), REG1A (1 µg/mL; Cat. No. ab47099), p53 (1:1000; Cat. No. ab32049), or GAPDH (1:10000; Cat. No. ab181602). Bands were visualized using enhanced chemiluminescence (Bio-Rad) and quantified using ImageJ software.

### 2.6 Methylated RNA immunoprecipitation followed by qRT–PCR

MeRIP assays were performed using the Magna MeRIP™ m^6^A kit (Millipore) according to the manufacturer’s instructions. Briefly, total RNA was extracted using TRIzol reagent, and 5 μg of total RNA was fragmented by heating at 94 °C for 5 min. Fragmented RNA was incubated overnight at 4 °C with an anti-m^6^A antibody or IgG as a negative control, both conjugated to magnetic beads. After extensive washing, m^6^A-enriched RNA was eluted, purified, reverse-transcribed, and quantified by qRT–PCR. The relative m^6^A enrichment of REG1A mRNA was calculated as the percentage of immunoprecipitated RNA relative to input RNA (IP/Input, %).

### 2.7 CCK-8 proliferation assay

Cells were seeded in 96-well plates at a density of 3 × 10³ cells per well. At indicated time points (0, 24, 48, and 72 hours), 10 μL of CCK-8 reagent (Dojindo) was added to each well and incubated for 2 hours at 37 °C. Absorbance was measured at 450 nm using a microplate reader. Each experimental group included five replicate wells.

### 2.8 Colony formation assay

For long-term proliferation assessment, cells were seeded at 400 cells per well in 6-well plates and cultured for 10–14 days with medium replaced every three days. Colonies were fixed with methanol for 15 minutes, stained with 0.1% crystal violet for 30 minutes, washed extensively with water, air-dried, photographed, and quantified using ImageJ. A colony was defined as a group of ≥50 cells.

### 2.9 mRNA stability assay

To assess REG1A mRNA stability, cells were treated with Actinomycin D (ActD; 5 μg/mL; Sigma-Aldrich) to block *de novo* transcription. Total RNA was harvested at 0, 2, 4, 6, and 8 hours after treatment and extracted using TRIzol reagent as described above. REG1A mRNA levels were quantified by RT-qPCR and normalized to GAPDH, which showed stable Ct values across all time points during ActD treatment. Relative mRNA abundance was calculated using the 2^−^ΔΔCt method and expressed as a percentage of the 0-hour value. The relative mRNA levels were fitted to a first-order exponential decay model: mRNA(t) = mRNA (0) × e^(−kt), using nonlinear regression analysis in GraphPad Prism 9, where k is the decay rate constant. Transcript half-life (t_1_/_2_) was calculated as t_1_ /_2_ = ln (2)/k. All experiments were performed in at least three independent biological replicates.

### 2.10 RNA immunoprecipitation assay and YTHDF2 knockdown

To investigate the interaction between YTHDF2 and REG1A mRNA, YTHDF2 expression was first silenced using small interfering RNA (siRNA). The following siRNA sequences were used: si-YTHDF2 (sense): 5′-CAAGGAAACAAAGTGCAAA-3′. A non-targeting scramble siRNA (si-NC) (sense): 5′-UUCUCCGAACGUGUCACGUTT-3′ was used as a negative control. Transfection was performed using Lipofectamine RNAiMAX (Thermo Fisher Scientific, Waltham, MA, USA) according to the manufacturer’s instructions. Cells were transfected at approximately 40–50% confluency, and experiments were conducted 48 hours post-transfection. Knockdown efficiency was confirmed at both the mRNA level by RT-qPCR and the protein level by Western blotting prior to subsequent functional assays. Only experiments achieving >70% knockdown efficiency at the protein level were included in downstream analyses. RIP assays were subsequently performed using a Magna RIP™ RNA-Binding Protein Immunoprecipitation Kit (Millipore) according to the manufacturer’s instructions with minor modifications. Briefly, cells were lysed in RIP lysis buffer supplemented with protease inhibitor cocktail and RNase inhibitor. For each immunoprecipitation reaction, lysate equivalent to approximately 1 × 10^7^ cells (corresponding to ~500 μg total protein) was used. An aliquot (10%) of the lysate was reserved as input control. Magnetic beads were pre-incubated with 5 μg of anti-YTHDF2 antibody (Abcam, ab246514) or normal rabbit IgG (Abcam, ab210849) as a negative control for 30 minutes at room temperature with rotation, followed by incubation with cell lysates overnight at 4 °C. After immunoprecipitation, the beads were washed six times with cold RIP wash buffer to minimize nonspecific binding. RNA–protein complexes were treated with proteinase K to remove proteins, and the co-immunoprecipitated RNA was purified by phenol–chloroform extraction according to the kit protocol. The enrichment of REG1A mRNA in YTHDF2 immunoprecipitates was quantified by RT-qPCR. RIP signals were normalized to input RNA and expressed as fold enrichment relative to IgG control, calculated as 2^(Ct_IgG − Ct_RIP), where both RIP and IgG values were first normalized to their respective input. RIP specificity was validated by demonstrating significant enrichment of REG1A mRNA in YTHDF2 immunoprecipitates compared with IgG negative control. In addition, YTHDF2 knockdown significantly reduced REG1A mRNA enrichment in RIP assays, further confirming the specificity of the observed interaction. All RIP experiments were performed in at least three independent biological replicates.

### 2.11 Chromatin immunoprecipitation assay

Chromatin immunoprecipitation (ChIP) assays were performed to evaluate tamoxifen-induced p53 binding to the ALKBH5 promoter using a ChIP assay kit (Millipore) according to the manufacturer’s instructions. Briefly, cells expressing wild-type or mutant p53 were treated with vehicle control (ethanol) or 1 μM 4-hydroxytamoxifen (4-OHT) for the indicated duration prior to crosslinking. Cells were crosslinked with 1% formaldehyde for 10 minutes at room temperature and quenched with 125 mM glycine. Cells were then lysed, and chromatin was isolated and sonicated to generate DNA fragments of approximately 200–500 bp. After centrifugation, the soluble chromatin fraction was collected, and 10% of the chromatin was reserved as input control. For immunoprecipitation, chromatin equivalent to approximately 1 × 10^6^ cells (corresponding to ~500 μg of DNA–protein complex) was incubated overnight at 4 °C with 5 μg of anti-p53 antibody (Cell Signaling Technology, #9282) or normal rabbit IgG (Cell Signaling Technology, #2729) as a negative control. Immune complexes were captured using protein A/G magnetic beads and washed sequentially with low-salt, high-salt, LiCl, and TE buffers. After reversal of crosslinks and proteinase K digestion, DNA was purified and subjected to quantitative PCR using primers flanking the predicted p53-binding site within the ALKBH5 promoter. A distal genomic region lacking predicted p53 response elements was amplified in parallel as a negative genomic control to confirm binding specificity. ChIP signals were normalized to input DNA and expressed as fold enrichment relative to IgG control.

### 2.12 Luciferase reporter assay

The ALKBH5 promoter region containing the predicted p53-binding site was cloned into the pGL3-Basic luciferase vector (Promega, E1751). A mutant construct disrupting the p53-binding site was generated by site-directed mutagenesis and synthesized by Sangon Biotech (Shanghai, China) to confirm binding specificity. Cells expressing wild-type or mutant p53 were co-transfected with firefly luciferase constructs and Renilla luciferase plasmid (pRL-TK, Promega, E2241) as an internal control using Lipofectamine™ 3000 (Invitrogen). Twenty-four hours post-transfection, cells were treated with vehicle or 1 μM 4-OHT for 24 hours. Luciferase activity was measured using the Dual-Luciferase Reporter Assay System (Promega, E1910). Firefly luciferase activity was normalized to Renilla luciferase to control for transfection efficiency. Baseline promoter activity was determined under vehicle-treated conditions, and the effect of 4-OHT on ALKBH5 promoter activity in wild-type versus mutant p53 backgrounds was calculated relative to the corresponding vehicle controls. All assays were performed in at least three independent biological replicates.

### 2.13 Statistical analysis

All data are presented as mean ± standard deviation (SD) from at least three independent biological replicates. For proliferation assays (CCK-8 and colony formation), each experiment included multiple technical replicates per biological replicate as indicated in the figure legends. Prior to statistical comparisons, data distribution was assessed for normality using the Shapiro–Wilk test, and homogeneity of variances was evaluated using Levene’s test. For comparisons between two groups, unpaired two-tailed Student’s t-tests were performed when assumptions of normality and equal variance were satisfied; otherwise, the Mann–Whitney U test was applied. For comparisons among three or more groups, one-way ANOVA followed by Tukey’s multiple comparison test was used when parametric assumptions were met; otherwise, the Kruskal–Wallis test with Dunn’s *post hoc* correction was applied. For ChIP, MeRIP, RIP, and mRNA stability assays, at least three independent biological replicates were analyzed, and appropriate parametric or non-parametric tests were selected based on the above criteria. All statistical analyses were conducted using GraphPad Prism 9 (GraphPad Software, San Diego, CA, USA). A two-sided P value < 0.05 was considered statistically significant.

## 3 Results

### 3.1 p53 differentially regulates tamoxifen-induced proliferation of immortalized endometrial cells depending on its mutational status

**Figure 1:**
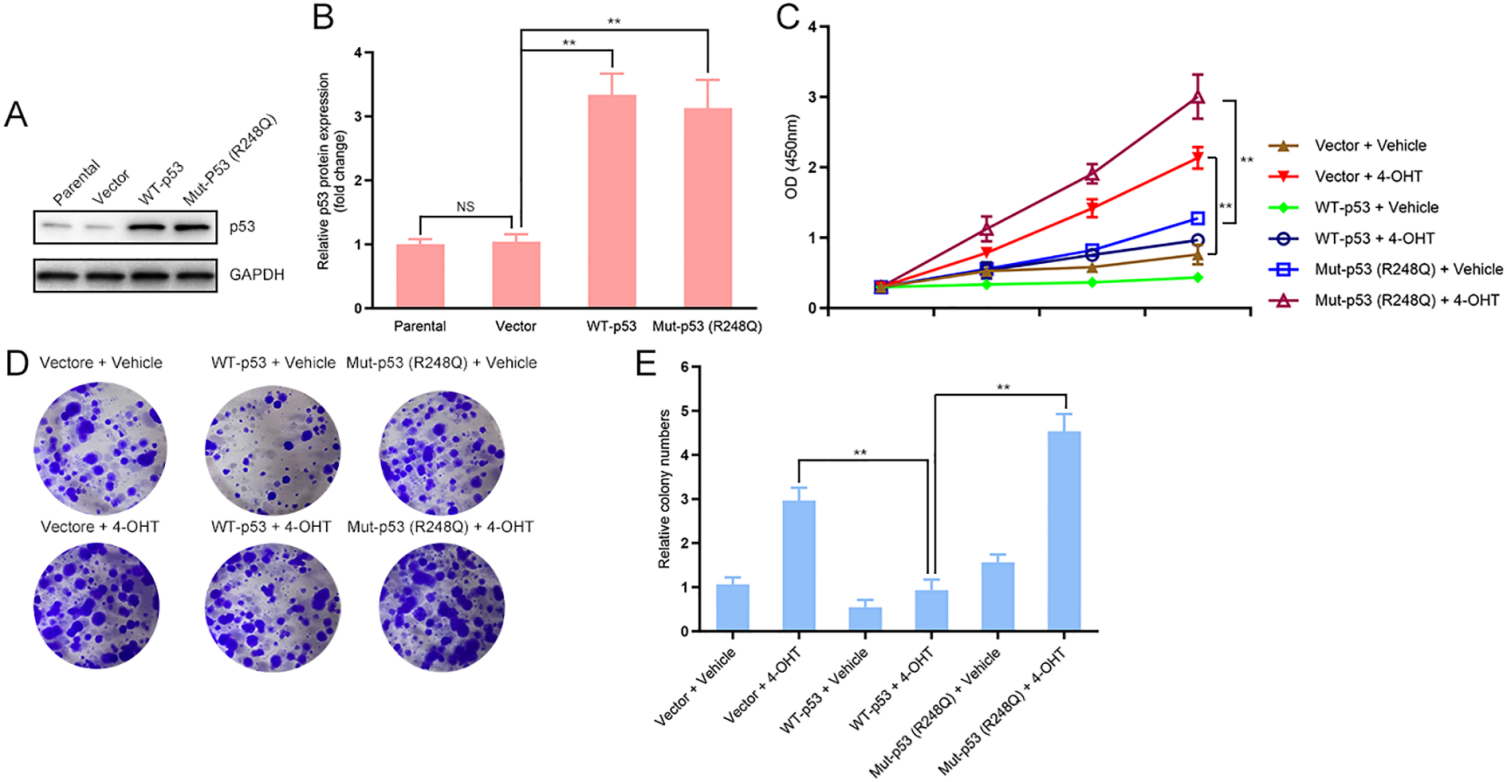
p53 differentially regulates tamoxifen-induced proliferation of immortalized endometrial epithelial cells depending on its mutational status. **(A, B)** Immortalized human endometrial epithelial cells were stably transduced with lentiviral vectors encoding wild-type (WT) p53, mutant p53 (R248Q), or empty vector control. Western blotting was performed to confirm p53 expression levels using anti-p53 antibodies, with GAPDH as the loading control. Densitometric quantification of protein bands was conducted using ImageJ software. **(C)** Cells were treated with 4-hydroxytamoxifen (4-OHT, 1 μM) or vehicle (ethanol) for 72 hours, and cell viability was measured using the CCK-8 assay. Absorbance was recorded at 450 nm using a microplate reader. **(D, E)** For colony formation assays, 400 cells per well were seeded in six-well plates and cultured for 14 days in the presence or absence of 4-OHT (1 μM). Colonies were fixed with methanol, stained with 0.1% crystal violet, and counted using ImageJ software. All experiments were independently repeated at least three times (n = 3 biological replicates per group). *P* < 0.01; NS, not significant.

To investigate the role of p53 in tamoxifen-induced endometrial cell proliferation, we first established stable cell lines by lentiviral transduction of wild-type (WT) p53 or mutant p53 (R248Q) into immortalized endometrial cells. Western blot analysis confirmed that both WT and R248Q p53 were efficiently overexpressed compared with control cells (**Figures 1A, B**). Next, we assessed the effects of p53 on cell proliferation in response to 4-hydroxytamoxifen (4-OHT). CCK-8 assays revealed that WT p53 significantly suppressed cell proliferation upon 4-OHT treatment, whereas R248Q mutant p53 enhanced proliferative activity compared with control cells (**Figure 1C**). Consistent with these findings, colony formation assays demonstrated that WT p53 reduced colony-forming ability under 4-OHT stimulation, while the R248Q mutant promoted colony formation (**Figures 1D, E**). Overall, these findings indicate that p53 plays a pivotal regulatory role in tamoxifen-induced endometrial cell proliferation, with WT p53 exerting an inhibitory effect and the R248Q mutant conferring a proliferative advantage.

### 3.2 Tamoxifen-induced REG1A expression restrains the proliferative response of endometrial epithelial cells

**Figure 2:**
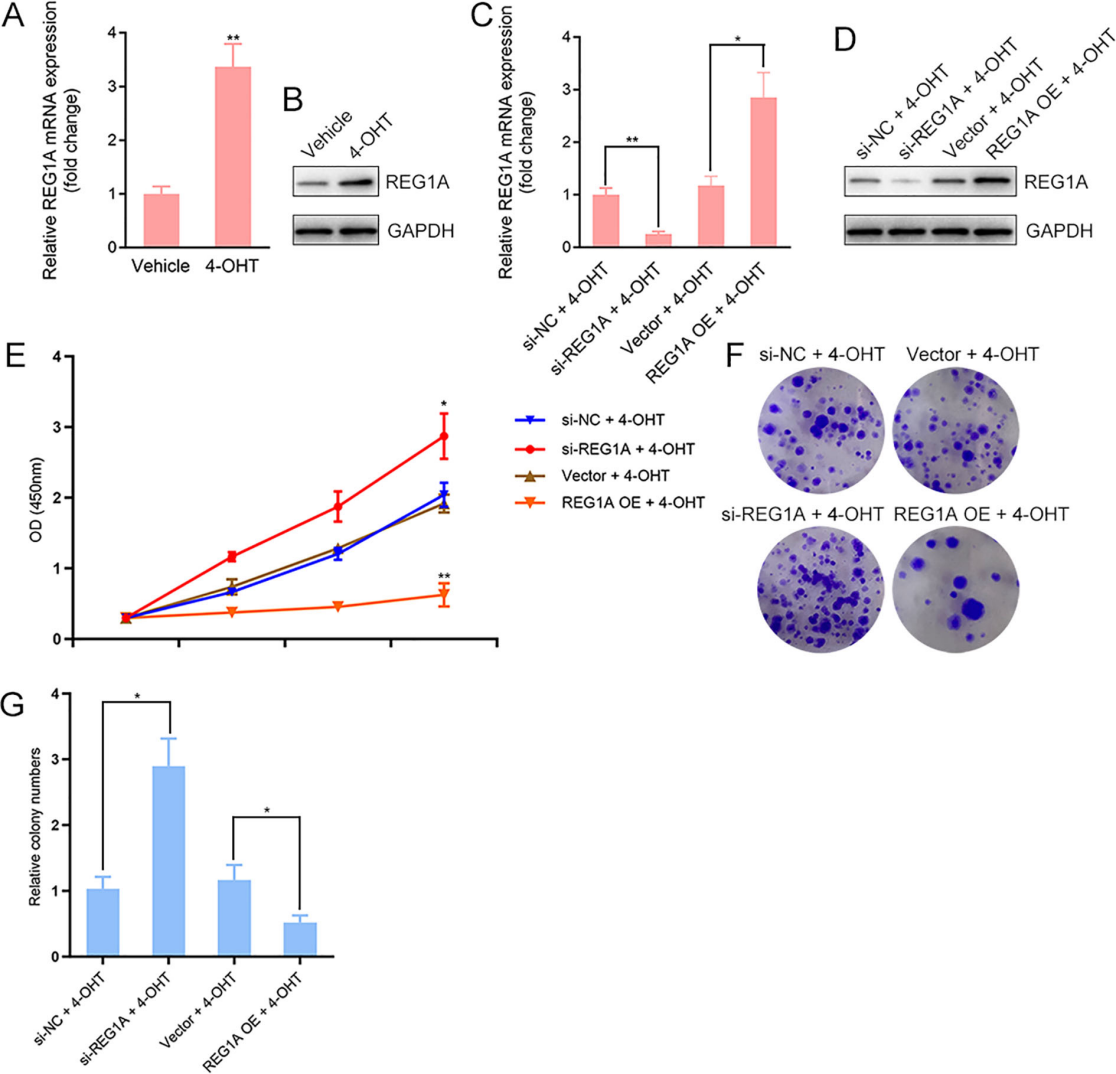
Tamoxifen induces REG1A expression and REG1A restrains endometrial epithelial cell proliferation. **(A)** Immortalized human endometrial epithelial cells were treated with 4-hydroxytamoxifen (4-OHT, 1μM) or vehicle (ethanol) for 48 h, and REG1A mRNA expression was quantified by qRT-PCR. **(B)** REG1A protein levels in cells treated with 4-OHT or vehicle were analyzed by Western blotting. **(C, D)** To examine the functional role of REG1A, cells were transfected with REG1A-targeting siRNA or stably transduced with lentiviral vectors expressing REG1A. Knockdown and overexpression efficiencies were validated by qRT-PCR and Western blotting. **(E)** Cell viability was assessed using the CCK-8 assay following 4-OHT treatment in REG1A-silenced or REG1A-overexpressing cells. **(F, G)** Colony formation assay showing the clonogenic capacity of REG1A-silenced or REG1A-overexpressing cells under 4-OHT treatment. *P* < 0.05, *P* < 0.01. OE, overexpression.

REG1A is a secreted C-type lectin-like protein essential for epithelial repair and homeostasis, yet its physiological role within the endometrium has not been clearly defined. To investigate whether REG1A participates in the proliferative response of endometrial epithelial cells to tamoxifen, immortalized human endometrial epithelial cells were treated with 4-hydroxytamoxifen (4-OHT, 1 μM) for 48 hours. Western blot and qRT-PCR analyses revealed a significant upregulation of REG1A expression at both the mRNA and protein levels following 4-OHT exposure compared with vehicle-treated controls (**Figures 2A, B**), suggesting that tamoxifen enhances REG1A expression through transcriptional or post-transcriptional mechanisms. To clarify the functional role of REG1A, cells were transfected with REG1A-targeting siRNA or stably transduced with lentiviral vectors encoding REG1A. Knockdown and overexpression efficiencies were verified by qRT-PCR and Western blot analysis (**Figures 2C, D**). CCK-8 assays demonstrated that REG1A silencing further augmented 4-OHT–induced cell proliferation, whereas REG1A overexpression significantly suppressed tamoxifen-stimulated cell viability compared with vector controls (**Figure 2E**). Consistent results were obtained in colony formation assays, where REG1A knockdown increased both the number and size of colonies following 4-OHT treatment, while REG1A overexpression markedly reduced clonogenic capacity (**Figures 2F, G**). Collectively, these findings indicate that tamoxifen induces REG1A expression, which functions as a negative feedback regulator to limit excessive endometrial epithelial cell proliferation.

### 3.3 Wild-type and mutant p53 differentially regulate REG1A expression in response to tamoxifen in endometrial cells

**Figure 3:**
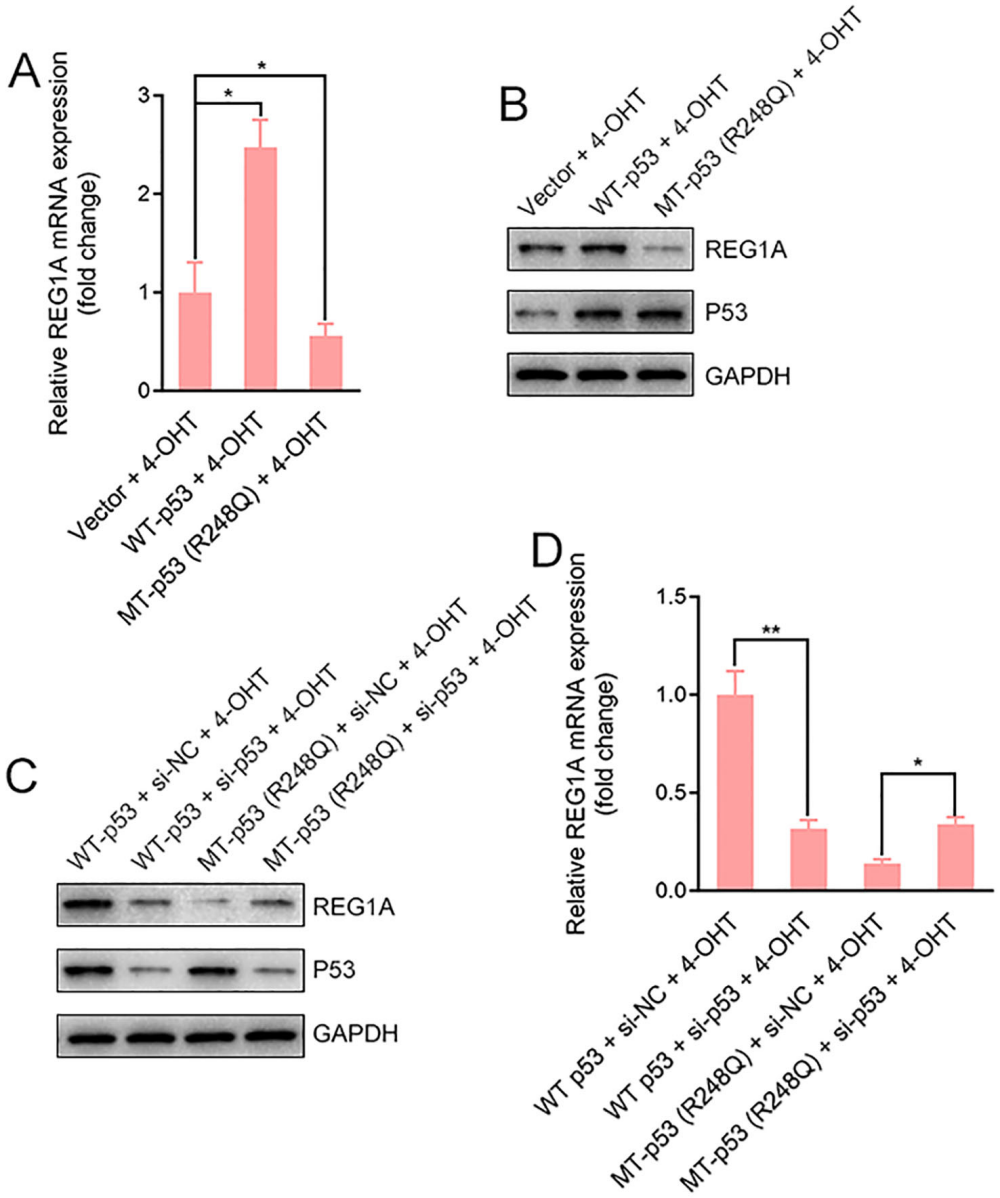
P53 mutational status determines REG1A expression in response to tamoxifen. **(A, B)** Immortalized human endometrial epithelial cells were stably transduced with lentiviral vectors expressing wild-type (WT) p53, mutant p53 (R248Q), or empty vector control. Cells were treated with 4-hydroxytamoxifen (4-OHT, 1 μM) for 48 h, and REG1A protein and mRNA levels were analyzed by Western blotting and qRT-PCR. **(C, D)** To confirm p53 dependence, cells were transfected with siRNA targeting p53 (si-p53) prior to 4-OHT treatment. Silencing of p53 abolished the differential regulation of REG1A at both the protein **(C)** and mRNA **(D)** levels induced by WT or mutant p53. *P* < 0.05, *P* < 0.01.

To determine whether p53 mutational status modulates tamoxifen-induced REG1A expression, immortalized human endometrial epithelial cells were stably transduced with lentiviral vectors expressing either wild-type (WT) p53 or the R248Q mutant and subsequently treated with 4-hydroxytamoxifen (4-OHT, 1 μM) for 48 hours. Western blotting and qRT-PCR analyses revealed that REG1A expression was markedly upregulated by 4-OHT in WT p53–expressing cells, whereas the same treatment significantly diminished REG1A expression in cells expressing mutant p53 (**Figures 3A, B**). To verify that these effects were directly mediated by p53, p53 expression was silenced using siRNA prior to 4-OHT stimulation. Loss of p53 eliminated both the enhancing effect of WT p53 and the suppressive effect of the R248Q mutant on REG1A expression (**Figures 3C, D**), confirming that REG1A regulation by tamoxifen is p53-dependent. These findings indicate that tamoxifen modulates REG1A expression in a p53 status–dependent manner: WT p53 activates REG1A expression, whereas the R248Q mutant fails to do so and instead represses REG1A, thereby altering the cellular proliferative response to tamoxifen.

### 3.4 Tamoxifen induces p53 binding to the ALKBH5 promoter, differentially regulating its transcription depending on p53 status

**Figure 4:**
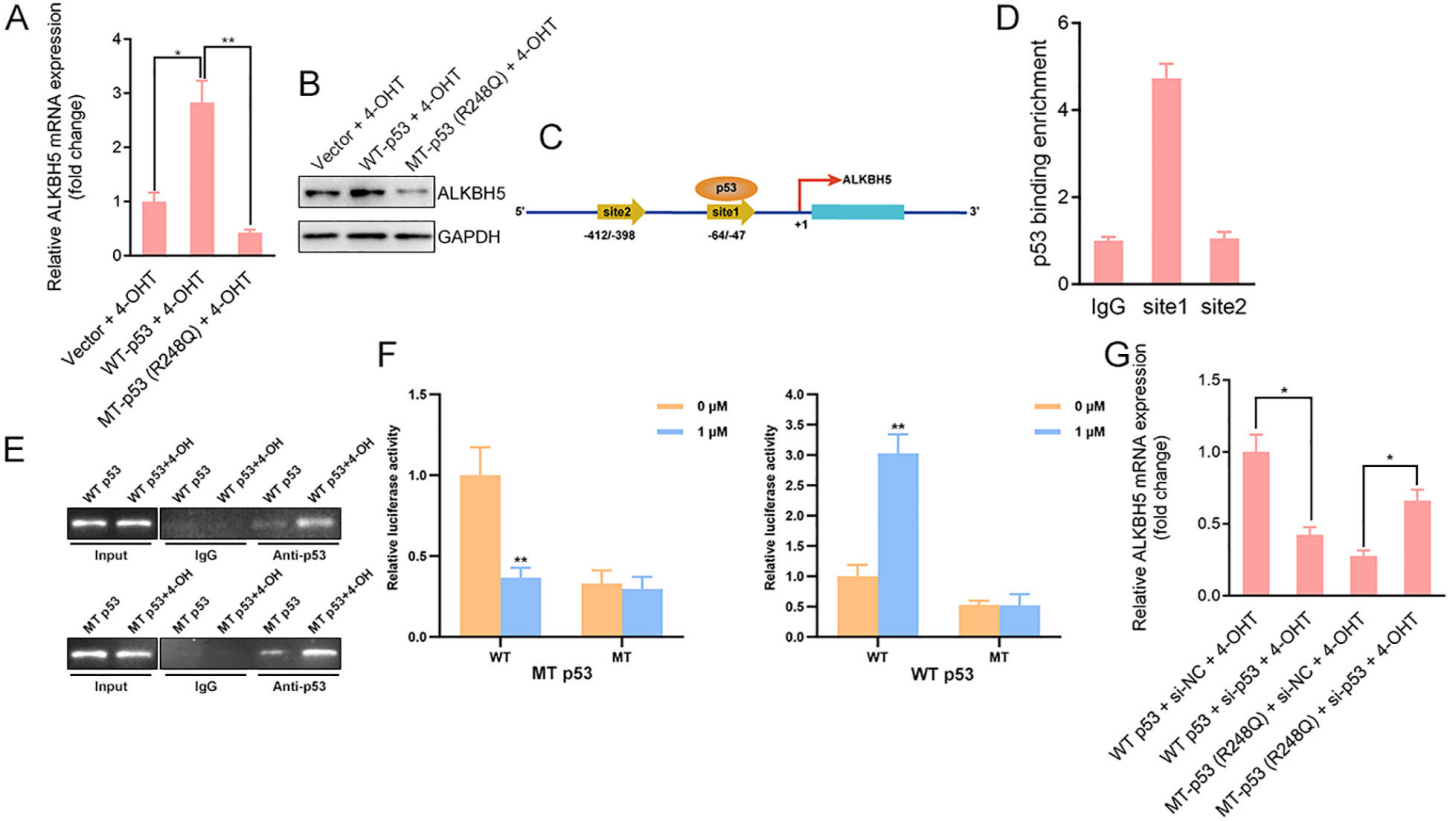
Tamoxifen induces p53 binding to the ALKBH5 promoter and regulates ALKBH5 transcription in a p53-allele–dependent manner. **(A, B)** Immortalized human endometrial epithelial cells stably expressing wild-type (WT) p53 or the R248Q mutant were treated with 4-hydroxytamoxifen (4-OHT, 1 μM) for 48 h. ALKBH5 mRNA **(A)** and protein **(B)** levels were analyzed by qRT-PCR and Western blotting, respectively. **(C)** Schematic representation of the ALKBH5 promoter showing predicted p53-binding sites identified by bioinformatic analysis. **(D)** Chromatin immunoprecipitation (ChIP) assays using anti-p53 antibodies were performed to assess 4-OHT–induced recruitment of WT or mutant p53 to the ALKBH5 promoter. **(E, F)** Luciferase reporter assays were performed using ALKBH5 promoter–driven luciferase constructs containing either the wild-type promoter or promoters with mutations in the predicted p53-binding sites. Cells expressing wild-type (WT) p53 or the R248Q mutant were treated with 4-hydroxytamoxifen (4-OHT) for 24 h. Firefly luciferase activity was normalized to Renilla luciferase activity. **(G)** Cells expressing WT or mutant p53 were transfected with control siRNA (si-NC) or p53-targeting siRNA (si-p53) prior to 4-OHT treatment. ALKBH5 mRNA levels were quantified by qRT-PCR to assess p53 dependency at the transcriptional level. Data are presented as mean ± SD from at least three independent experiments. *P* < 0.05, *P* < 0.01.

Previous studies have shown that ALKBH5 functions as a key m^6^A demethylase in p53-related tumor regulation (29, 30). Immortalized human endometrial epithelial cells stably expressing wild-type (WT) p53 or the R248Q mutant were treated with 4-hydroxytamoxifen (4-OHT, 1 μM) for 24 or 48 hours. qRT-PCR and Western blot analyses showed that 4-OHT markedly increased ALKBH5 mRNA and protein levels in WT p53-expressing cells, whereas ALKBH5 expression was significantly reduced in cells expressing the R248Q mutant (**Figures 4A, B**). Bioinformatic analysis identified two putative p53-binding sites within the ALKBH5 promoter (**Figure 4C**). Chromatin immunoprecipitation assays demonstrated that 4-OHT induced recruitment of both WT and mutant p53 to the ALKBH5 promoter (**Figure 4D**). Despite comparable promoter binding, luciferase reporter assays revealed opposing transcriptional outcomes: WT p53 significantly enhanced, whereas the R248Q mutant suppressed, ALKBH5 promoter activity following 4-OHT treatment (**Figures 4E, F**). Furthermore, siRNA-mediated depletion of p53 abolished both the transcriptional activation mediated by WT p53 and the repression imposed by mutant p53, as assessed by ALKBH5 mRNA expression (**Figure 4G**). Together, these results demonstrate that tamoxifen induces p53 binding to the ALKBH5 promoter, resulting in divergent transcriptional regulation of ALKBH5 depending on p53 status.

### 3.5 ALKBH5 stabilizes REG1A mRNA through m^6^A–YTHDF2–dependent regulation

**Figure 5:**
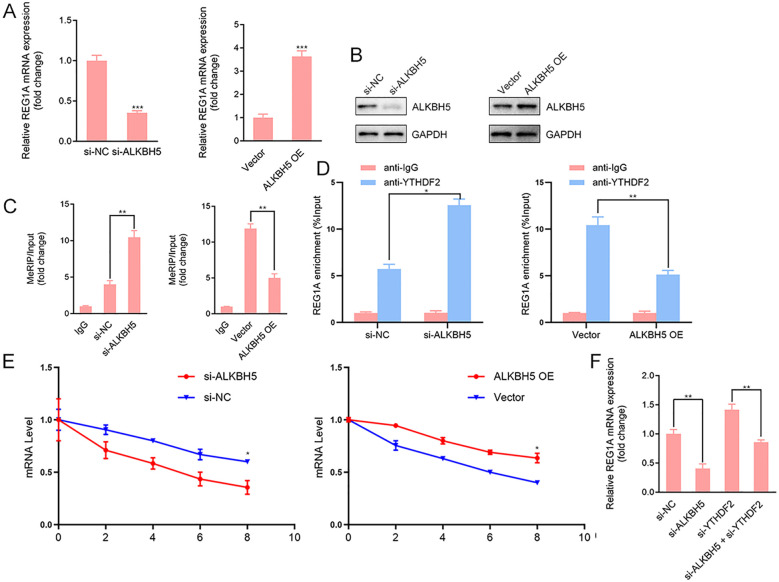
ALKBH5 stabilizes REG1A mRNA by removing m^6^A modifications and preventing YTHDF2-mediated decay. **(A, B)** Immortalized human endometrial epithelial cells were subjected to ALKBH5 knockdown or overexpression. ALKBH5 expression efficiency was confirmed by qRT-PCR and Western blot analysis. REG1A mRNA and protein levels were quantified by qRT-PCR **(A)** and Western blot **(B)**, respectively. **(C)** m^6^A enrichment on REG1A mRNA was assessed by methylated RNA immunoprecipitation (MeRIP) followed by qPCR using an anti-m^6^A antibody. **(D)** RNA immunoprecipitation (RIP) assays using an anti-YTHDF2 antibody were performed to evaluate the interaction between YTHDF2 and REG1A mRNA. **(E)** REG1A mRNA stability was determined by Actinomycin D assay (5 μg/mL), with transcript levels measured at the indicated time points. **(F)** YTHDF2 was co-silenced in ALKBH5-knockdown cells, and REG1A mRNA levels were quantified by qRT-PCR to assess the contribution of YTHDF2 to REG1A mRNA decay. *P* < 0.05, *P* < 0.01, *P* < 0.001.

Mounting evidence indicates that ALKBH5, a major m^6^A RNA demethylase, regulates gene expression by modulating N^6^-methyladenosine marks, thereby influencing mRNA processing, stability, and translation (15, 31). To determine whether ALKBH5 regulates REG1A expression through an m^6^A-dependent post-transcriptional mechanism, immortalized human endometrial epithelial cells were subjected to ALKBH5 knockdown or overexpression. Quantitative RT–PCR and Western blot analyses revealed that ALKBH5 silencing significantly reduced, whereas ALKBH5 overexpression markedly increased, REG1A mRNA and protein levels (**Figures 5A, B**), indicating a positive regulatory role of ALKBH5 on REG1A expression. To assess whether this regulation involves m^6^A modification, methylated RNA immunoprecipitation (MeRIP)-qPCR was performed. ALKBH5 depletion led to increased m^6^A enrichment on REG1A mRNA, whereas ALKBH5 overexpression resulted in a pronounced reduction of m^6^A levels (**Figure 5C**), demonstrating that REG1A is a direct demethylation target of ALKBH5. Given that m^6^A marks frequently promote mRNA decay through recruitment of m^6^A readers, we next examined the involvement of YTHDF2, a key mediator of m^6^A-dependent mRNA degradation. RNA immunoprecipitation assays showed that YTHDF2 binds REG1A mRNA, and this interaction was enhanced upon ALKBH5 knockdown but attenuated by ALKBH5 overexpression (**Figure 5D**). Consistently, actinomycin D chase experiments demonstrated that ALKBH5 depletion significantly shortened the half-life of REG1A mRNA, whereas ALKBH5 overexpression prolonged its stability (**Figure 5E**). Importantly, co-silencing of YTHDF2 largely rescued REG1A mRNA stability in ALKBH5-deficient cells (**Figure 5F**), indicating that YTHDF2 is responsible for the accelerated decay of hypermethylated REG1A transcripts. Collectively, these findings establish that ALKBH5 stabilizes REG1A mRNA by removing m^6^A modifications and thereby preventing YTHDF2-mediated mRNA degradation.

### 3.6 A p53-dependent ALKBH5-REG1A axis governs the tamoxifen-induced proliferative response in endometrial epithelial cells

**Figure 6:**
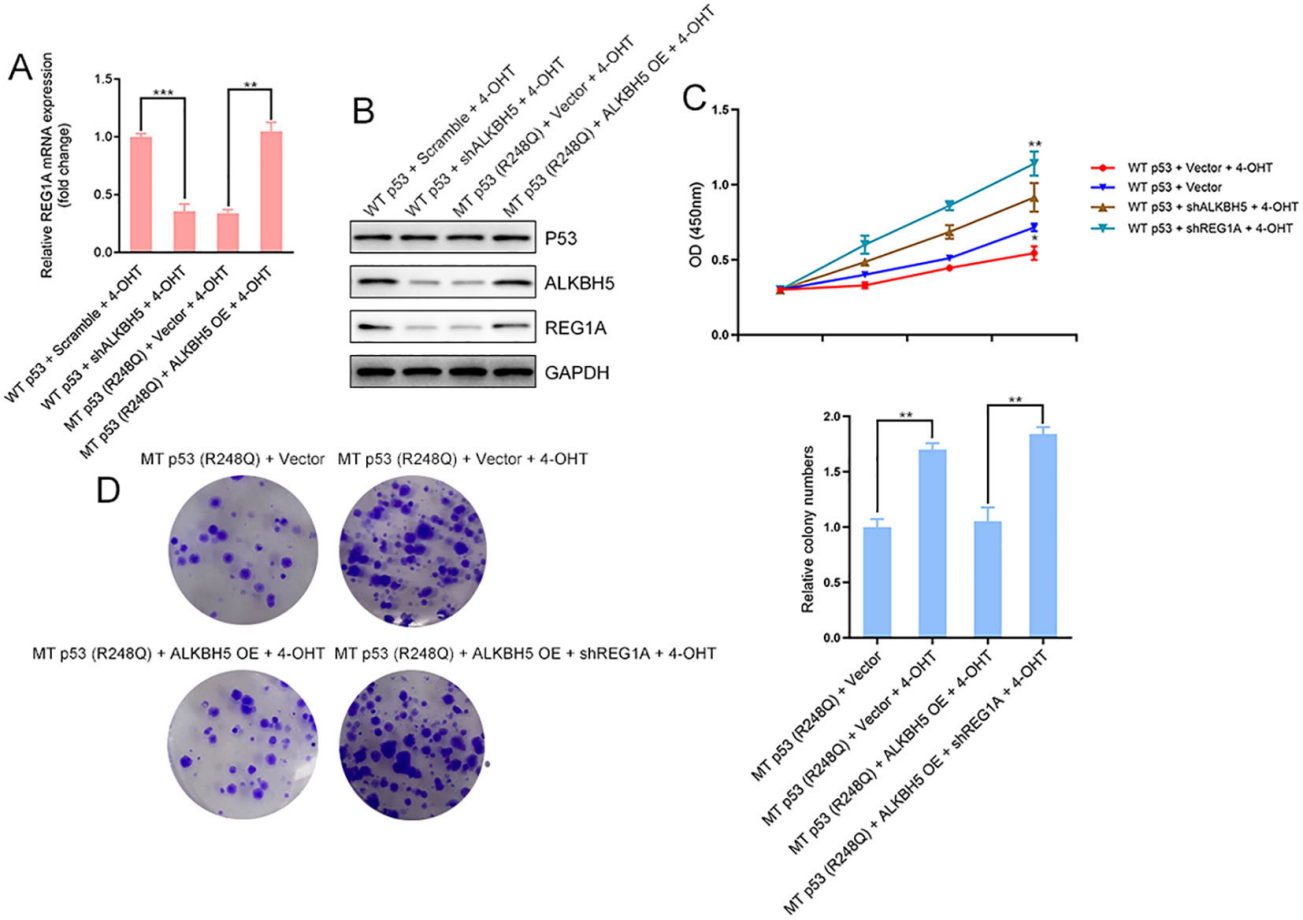
Tamoxifen regulates endometrial epithelial cell proliferation through a p53-dependent ALKBH5–REG1A axis. **(A, B)** Western blot and qRT-PCR analyses of ALKBH5, p53, and REG1A expression in immortalized human endometrial epithelial cells expressing wild-type (WT) or mutant p53 (R248Q). Cells were further subjected to ALKBH5 knockdown (WT p53 + ALKBH5 shRNA) or ALKBH5 overexpression (R248Q p53 + ALKBH5 OE), as indicated, and treated with vehicle or 4-hydroxytamoxifen (4-OHT, 1 μM) for 48 h. **(C, D)** Cell proliferation was assessed by CCK-8 assays, and long-term clonogenic capacity was evaluated by colony formation assays under the indicated genetic manipulations and 4-OHT treatment. ALKBH5 and REG1A knockdown or overexpression was achieved using lentiviral shRNA or overexpression constructs. Data are presented as mean ± SD from three independent experiments. *P* < 0.05, *P* < 0.01, *P* < 0.001.

To define the functional significance of the tamoxifen–p53–ALKBH5–REG1A regulatory cascade in endometrial epithelial cell proliferation, immortalized human endometrial epithelial cells were subjected to a series of genetic manipulations involving wild-type (WT) or mutant p53 (R248Q), in combination with ALKBH5 or REG1A knockdown or overexpression, followed by 4-hydroxytamoxifen (4-OHT, 1 μM) treatment. qRT-PCR and Western blot analyses revealed that 4-OHT markedly induced ALKBH5 and REG1A expression in WT p53–expressing cells, whereas both genes were significantly downregulated in cells expressing mutant p53 (**Figures 6A, B**). In WT p53–expressing cells, silencing ALKBH5 abolished the tamoxifen-induced upregulation of REG1A, and knockdown of REG1A further potentiated 4-OHT–stimulated cell proliferation, as determined by CCK-8 and colony formation assays (**Figures 6C, D**), indicating that REG1A functions downstream of ALKBH5 to restrain proliferative responses. In contrast, in R248Q mutant p53–expressing cells, enforced ALKBH5 expression restored REG1A levels to those observed in WT p53 cells and significantly attenuated tamoxifen-induced proliferation. This inhibitory effect was reversed upon concomitant REG1A depletion, confirming that the antiproliferative activity of ALKBH5 is mediated through REG1A upregulation (**Figures 6C, D**). Together, these findings demonstrate that tamoxifen regulates endometrial epithelial cell proliferation through a p53-dependent ALKBH5–REG1A axis. While WT p53 enables tamoxifen-induced activation of ALKBH5, promoting m^6^A demethylation and stabilization of REG1A mRNA to restrain cell proliferation, mutant p53 fails to induce ALKBH5, resulting in reduced REG1A expression and exaggerated proliferative responses.

## 4 Discussion

**Figure 7:**
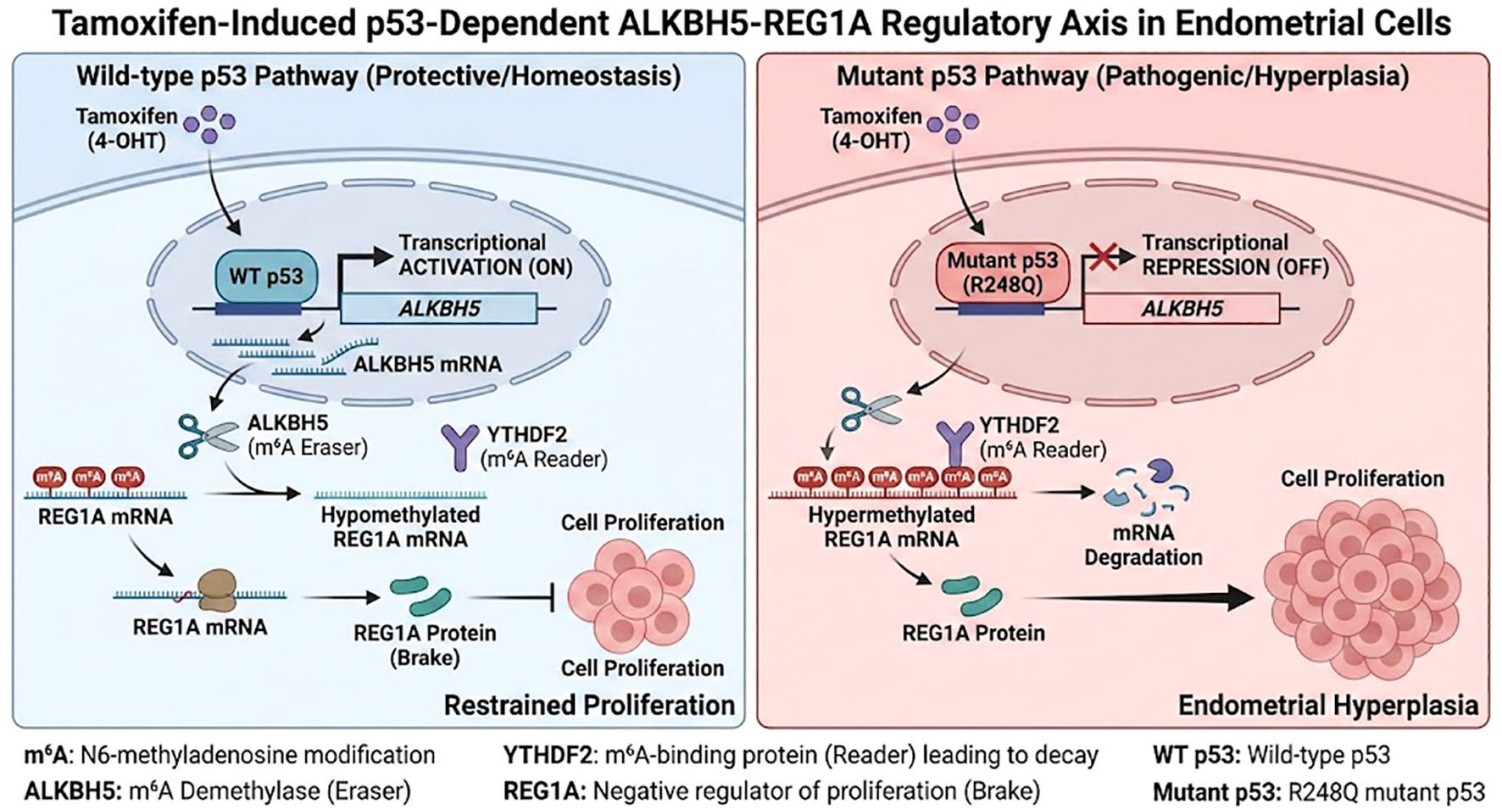
Schematic illustration of the p53-dependent mechanism governing endometrial responses to tamoxifen. (Left) In the presence of wild-type (WT) p53, tamoxifen stimulation promotes the recruitment of WT p53 to the ALKBH5 promoter, thereby transcriptionally activating ALKBH5. Upregulation of the m^6^A demethylase ALKBH5 facilitates the removal of m^6^A modifications from REG1A mRNA, preventing YTHDF2-mediated mRNA decay. Stabilization of REG1A leads to increased protein abundance, which functions as a negative feedback regulator to constrain endometrial cell proliferation. (Right) In contrast, mutant p53 (R248Q) fails to transactivate ALKBH5 in response to tamoxifen. The resultant reduction in ALKBH5 expression causes increased m^6^A methylation of REG1A mRNA, enhancing its recognition and degradation by YTHDF2. Loss of REG1A expression abolishes this proliferative restraint, ultimately driving tamoxifen-induced endometrial hyperplasia.

Tamoxifen remains the cornerstone of endocrine therapy for estrogen receptor-positive breast cancer; however, its estrogenic effects on the endometrium present a major clinical paradox. Prolonged tamoxifen treatment increases the risk of endometrial hyperplasia and carcinoma, particularly in postmenopausal women, yet the molecular mechanisms underlying this tissue-specific adverse effect remain poorly understood. Here, we identify a tamoxifen-p53-ALKBH5-REG1A signaling axis that governs endometrial epithelial cell proliferation in a p53 status-dependent manner (**Figure 7**).

Our results demonstrate that tamoxifen upregulates ALKBH5 and REG1A expression through a wild-type (WT) p53-dependent mechanism, whereas in cells harboring the R248Q mutant p53, both genes are downregulated, leading to enhanced proliferation. These findings are consistent with growing evidence that p53 status modulates m^6^A RNA methylation machinery and transcript stability (32, 33). Previous studies have shown that p53 transcriptionally regulates ALKBH5 expression, linking tumor suppressor pathways with the epitranscriptomic landscape (29, 34). Mechanistically, we confirmed that tamoxifen enhances p53 binding to the ALKBH5 promoter in WT but not mutant contexts, promoting ALKBH5 transcriptional activation. Elevated ALKBH5 demethylates m^6^A residues on REG1A mRNA, thereby reducing YTHDF2-mediated degradation and stabilizing REG1A expression. In mutant p53-expressing cells, this regulatory loop collapses-ALKBH5 expression decreases, REG1A transcripts become hypermethylated, and their stability declines-resulting in excessive proliferative signaling. This defines a previously unrecognized epitranscriptomic checkpoint that integrates hormonal signaling, p53 activity, and RNA modification dynamics.

Our findings extend prior studies on ALKBH5 in reproductive and oncogenic contexts. ALKBH5 is frequently upregulated in endometrial cancer and promotes proliferation through IGF1R stabilization (18) Similarly, ALKBH5 promotes tumor progression in glioblastoma, colon, and ovarian cancers (35–37), whereas in certain settings, it functions as a tumor suppressor (15). Such bidirectional effects are consistent with recent insights that m^6^A modifiers act as context-dependent regulators of cellular growth and stress responses (15).

Interestingly, REG1A, though often implicated in oncogenic roles in colorectal and bladder cancers (24, 38), has been more broadly reported as a pro-proliferative or oncogenic factor in several malignancies, including gastrointestinal and pancreatic cancers, where it promotes cell growth, survival, and tumor progression (25, 26, 39, 40). However, accumulating evidence suggests that the biological effects of REG family members may be highly context-dependent, varying across tissue types and microenvironmental conditions. For example, in gastric cancer, REG1A expression is significantly downregulated, and its overexpression suppresses cell invasion and proliferation while promoting apoptosis (41). Similarly, REG3A, another member of the REG family, exerts growth-inhibitory effects in gastric cancer, reducing proliferation and invasion and enhancing apoptosis (42). These studies support the notion that REG family proteins can function as negative regulators of cell growth in certain epithelial contexts. Consistent with this notion, REG1A appears to exert a growth-limiting effect in the endometrial epithelium in the present study. In our model, REG1A operates downstream of ALKBH5 as a negative feedback regulator, constraining tamoxifen-induced hyperproliferation. This apparent discrepancy relative to its oncogenic roles in other cancer types may reflect differences in cellular lineage, hormonal signaling context, and upstream transcriptional regulation. In hormone-responsive tissues such as the endometrium, REG1A may participate in stress-adaptive or differentiation-associated programs rather than classical oncogenic pathways. These findings highlight the tissue- and context-specific nature of REG1A function, caution against generalizing its oncogenic role to hormone-responsive epithelial tissues, and underscore the functional plasticity of regeneration-associated genes in maintaining endometrial homeostasis.

Clinically, these findings provide a mechanistic rationale for variable susceptibility to tamoxifen-associated endometrial pathology. In women with p53 mutations, the loss of ALKBH5-REG1A-mediated negative feedback may predispose to hyperplasia. Thus, p53 mutational profiling could inform individualized endocrine therapy decisions. Moreover, pharmacologic activation of ALKBH5 or stabilization of REG1A transcripts could mitigate uterine side effects while maintaining tamoxifen’s anticancer efficacy. Such approaches are conceptually supported by recent work highlighting the therapeutic potential of targeting m^6^A dynamics (43, 44). However, it should be emphasized that these clinical implications are hypothesis-generating and based solely on *in vitro* evidence. Further validation in animal models and clinical specimens will be required to determine whether p53 mutational status can serve as a predictive biomarker or whether targeting the ALKBH5–REG1A pathway holds genuine therapeutic relevance.

Despite these advances, several limitations should be acknowledged. This study employed immortalized endometrial epithelial cells and focused on a single p53 gain-of-function mutant, R248Q. This mutant was selected on the basis of its high prevalence in endometrial carcinoma and its well-established capacity to actively reprogram transcriptional networks, making it a clinically relevant model for investigating tamoxifen-associated endometrial pathology. Nevertheless, other hotspot mutants such as R175H or R273H may exert distinct regulatory effects, and the extent to which the ALKBH5–REG1A axis represents a generalizable mechanism across different p53 mutational contexts remains to be determined. Future studies using *in vivo* or organoid models encompassing diverse p53 variants are therefore necessary to confirm the universality of this regulatory axis. In addition, the downstream effectors linking REG1A to specific cell cycle regulators warrant further exploration.

## 5 Conclusions

In summary, we delineate a tamoxifen-p53-ALKBH5-REG1A axis that couples hormonal signaling to m^6^A-dependent transcript stabilization and endometrial proliferation. The dependence of this pathway on p53 mutational status provides a plausible molecular explanation for divergent endometrial responses to tamoxifen. While these findings are based on *in vitro* models and remain to be validated *in vivo*, they suggest that p53 mutational status may represent a potential avenue for biomarker-guided strategies to prevent tamoxifen-induced endometrial hyperplasia, warranting further investigation in clinical and animal model settings.

## Data Availability

The raw data supporting the conclusions of this article will be made available by the authors, without undue reservation.
